# Hypothyroidism and hypopituitarism as immune-related adverse events due to lenvatinib plus pembrolizumab therapy in the immediate postoperative period after laparoscopic hepatectomy for liver metastases from gastric cancer: a case report

**DOI:** 10.1186/s40792-021-01346-w

**Published:** 2021-12-20

**Authors:** Kimimasa Sasaki, Shin Kobayashi, Masashi Kudo, Motokazu Sugimoto, Shinichiro Takahashi, Yoshiaki Nakamura, Akihito Kawazoe, Kohei Shitara, Takahiro Kinoshita, Naoto Gotohda

**Affiliations:** 1 Department of Hepatobiliary and Pancreatic Surgery, National Cancer Center Hospital East, 6-5-1, Kashiwanoha, Kashiwa-City, Chiba 277-8577 Japan; 2 Department of Gastrointestinal Oncology, National Cancer Center Hospital East, 6-5-1, Kashiwanoha, Kashiwa-City, Chiba 277-8577 Japan; 3 Department of Gastric Surgery, National Cancer Center Hospital East, 6-5-1, Kashiwanoha, Kashiwa-City, Chiba 277-8577 Japan

**Keywords:** Gastric cancer, Hypothyroidism, Hypopituitarism, Immune-related adverse events, Laparoscopic hepatectomy, Lenvatinib, Liver metastases, Pembrolizumab

## Abstract

**Background:**

Immune checkpoint inhibitors (ICIs) are emerging agents used for the treatment of various malignant tumors. As ICIs are generally used for unresectable malignant tumors, there have been only a few reports of patients who underwent surgery after receiving these drugs. Therefore, it remains unclear how immune-related adverse events (irAEs) affect the postoperative course. Here, we report a patient with advanced gastric cancer who underwent laparoscopic hepatectomy for liver metastases after an objective response with lenvatinib plus pembrolizumab and developed hypothyroidism and hypopituitarism as irAEs in the immediate postoperative period.

**Case presentation:**

A 73-year-old man had undergone total gastrectomy for pT4aN2M0 gastric cancer followed by adjuvant chemotherapy with S-1 and docetaxel, and developed liver metastases in segments 6 and 7. He was enrolled in phase 2 clinical trial of lenvatinib plus pembrolizumab. He continuously achieved a partial response with the study treatment, and the liver metastases were decreased in size on imaging. The tumors were judged to be resectable and the patient underwent laparoscopic partial hepatectomy for segments 6 and 7. From the 1st postoperative day, the patient continuously presented with fever and general fatigue, and his fasting blood glucose level remained slightly lower than that before the surgery. On the 4th postoperative day, laboratory examination revealed hypothyroidism and hypopituitarism, which were suspected to be irAE caused by lenvatinib plus pembrolizumab after surgery. He received hydrocortisone first, followed by levothyroxine after adrenal insufficiency was recovered. Subsequently, his fever, general fatigue, and any abnormality regarding fasting blood glucose level resolved, and he was discharged on the 12th postoperative day. After discharge, his laboratory data for thyroid and pituitary function remained stable while receiving hydrocortisone and levothyroxine without recurrence of gastric cancer.

**Conclusion:**

We present a case of laparoscopic hepatectomy after receiving lenvatinib plus pembrolizumab and developed hypothyroidism and hypopituitarism after surgery. Regarding surgery after ICI therapy, it is important to recognize that irAEs might occur in the postoperative period.

## Background

Immune checkpoint inhibitors (ICIs) are emerging agents used for the treatment of various malignant tumors. ICIs are different from conventional anti-cancer drugs in terms of notable side effects called immune-related adverse events (irAEs) such as colitis, hepatitis, and endocrinopathy, which might occur during treatment. As ICIs are generally used for unresectable malignant tumors, there have been only a few reports of patients who underwent surgery after receiving these drugs. Therefore, it remains unclear how irAEs affect the postoperative course.

Recently, a combination of lenvatinib, a small-molecule tyrosine kinase inhibitor, plus pembrolizumab, an anti-programed death-ligand 1 inhibitor, was shown to have significant antitumor activity with acceptable safety profiles for solid tumors in clinical trials, which led to accelerated approval by the U.S. Food and Drug Administration for the treatment of patients with endometrial cancer with a response rate of 40% [1]. Most recently, we reported promising results of a phase 2 study of lenvatinib plus pembrolizumab for advanced gastric cancer, with a response rate of 69% in the first- or second-line setting [2].

Here, we report a case of advanced gastric cancer who underwent laparoscopic hepatectomy for liver metastases after an objective response with lenvatinib plus pembrolizumab and developed hypothyroidism and hypopituitarism as irAEs in the immediate postoperative period. Because of the retrospective nature of the study and the absence of invasive interventions, patients’ personal written consents were waived.

## Case presentation

**Fig. 1:**
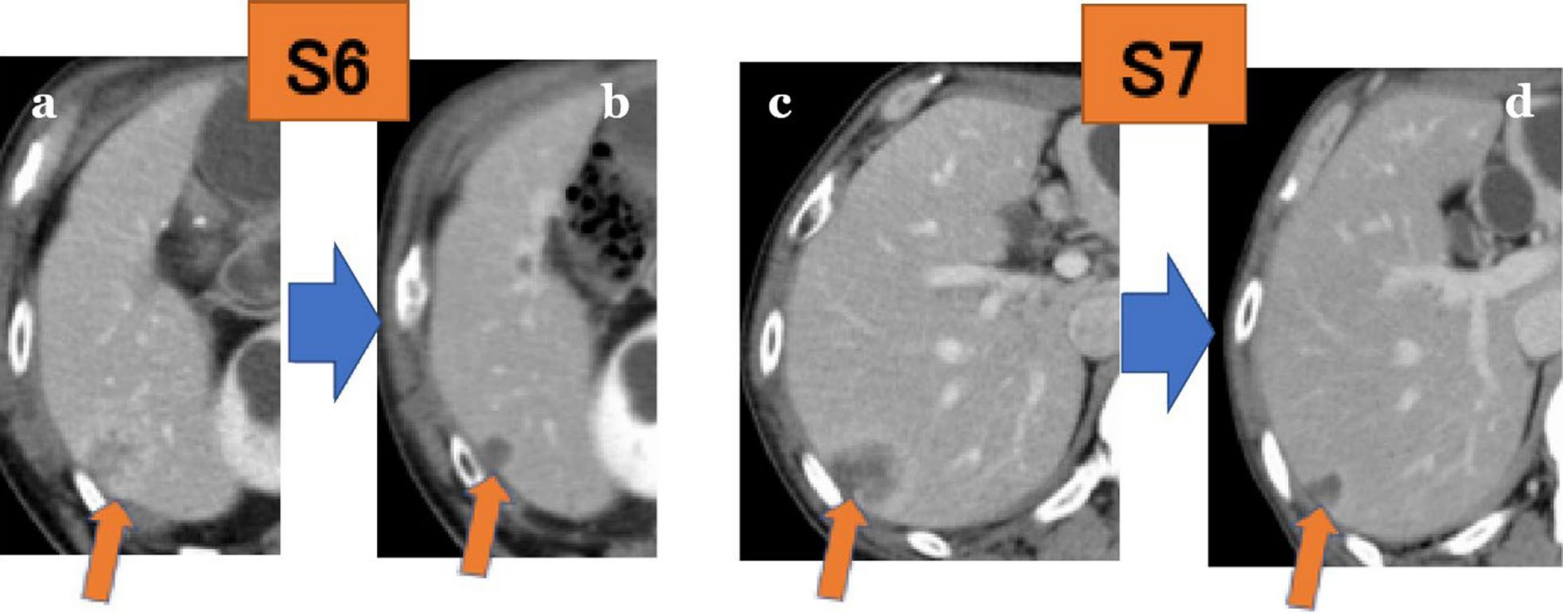
Computed tomography images before and after lenvatinib plus pembrolizumab treatment. (orange arrow) **a** Metastatic tumor in segment 6 before treatment. **b** Metastatic tumor in segment 6 after treatment. **c** Metastatic tumor in segment 7 before treatment. **d** Metastatic tumor in segment 7 after treatment

A 73-year-old man who had undergone total gastrectomy for pathological T4aN2M0 gastric cancer according to UICC-TNM classification 8th edition [3] followed by adjuvant chemotherapy with S-1 and docetaxel for 10 months developed liver metastases 1 year after gastrectomy. The tumors were located in segment 6 (17 mm in diameter) and segment 7 (27 mm in diameter). He was then enrolled in a phase 2 clinical trial of lenvatinib plus pembrolizumab and received the study treatment (20 mg oral lenvatinib once daily and 200 mg intravenous pembrolizumab every 3 weeks) for 5 months [4]. He had grade 2 oral mucositis and proteinuria according to the National Cancer Institute Common Terminology Criteria for Adverse Events (CTCAE version 4.03) during the study treatment [4]. Thyroid function tests revealed transient hyperthyroidism, which promptly returned to normal. He continuously achieved a partial response with the study treatment, and liver metastases were decreased in size to 8 mm in segments 6 and 11 mm in segment 7 (Fig. 1).

**Fig. 2:**
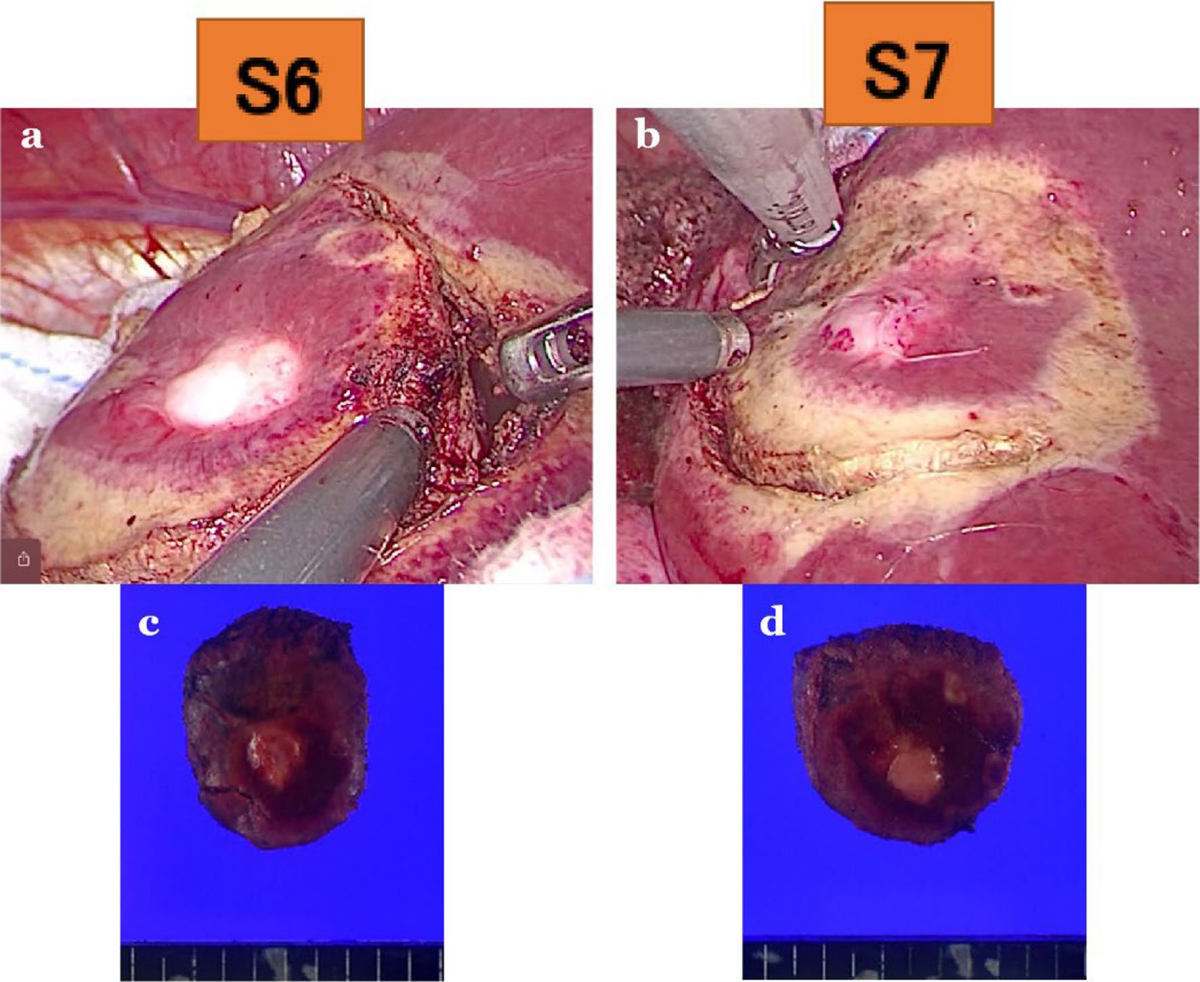
Intraoperative views of laparoscopic partial hepatectomy and liver specimens. **a** Metastatic tumor in segment 6. **b** Metastatic tumor in segment 7. **c** Metastatic tumor in segment 6. **d** Metastatic tumor in segment 7

Since the tumors were judged to be systemically under control due to decrease in size of liver metastases and no new lesions by positron emission tomography/computed tomography images, they came to be considered resectable. Four weeks after the last dose of pembrolizumab and 2 weeks after the last dose of lenvatinib, the patient underwent laparoscopic partial hepatectomy for segments 6 and 7. The operation time was 152 min, and blood loss was 109 mL (Fig. 2).

**Fig. 3:**
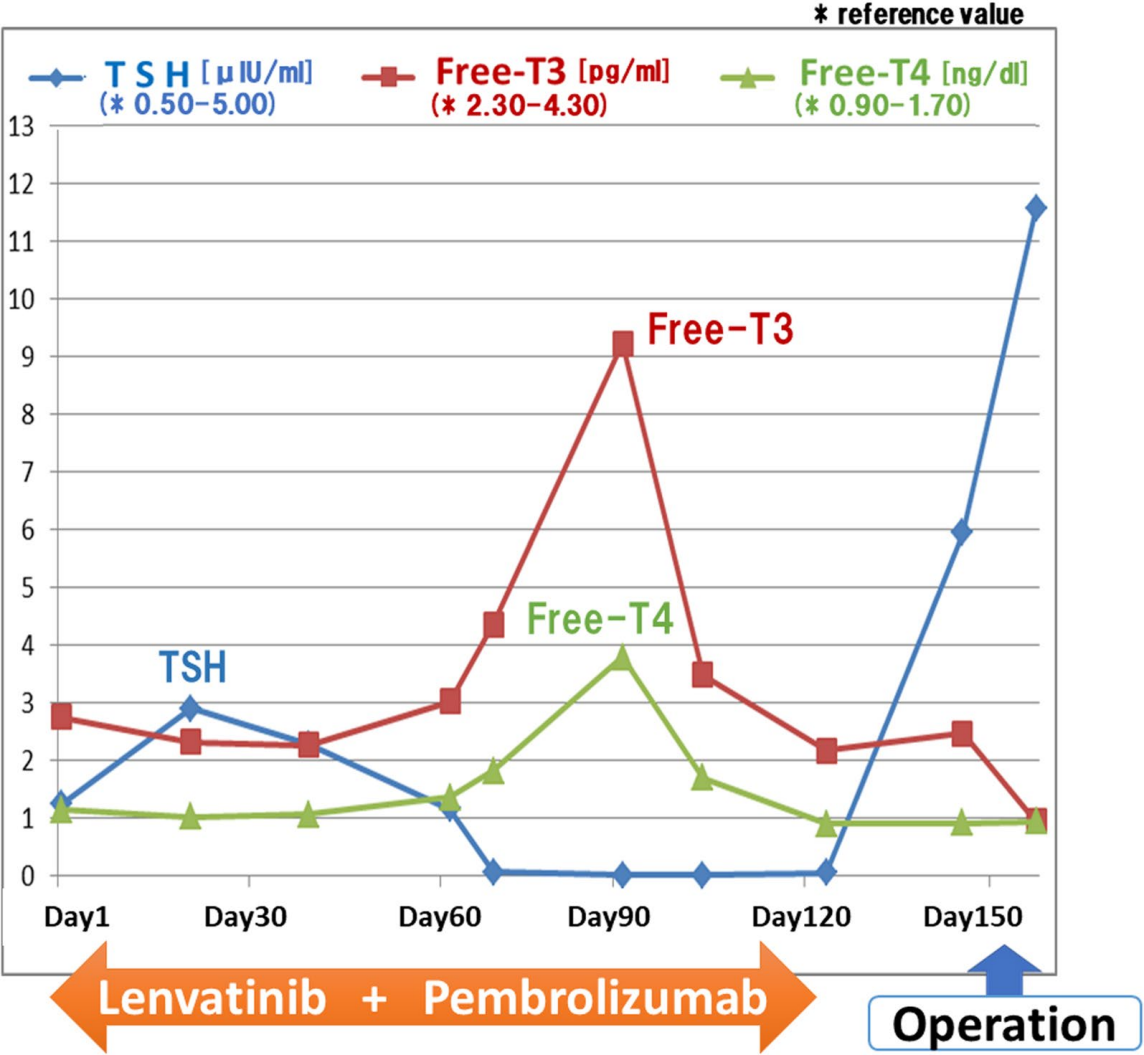
Transition of Thyroid-stimulating hormone (TSH), Free-T3 and Free-T4 across lenvatinib plus pembrolizumab treatment and operation

On the 1st postoperative day, his body temperature was elevated to 40.4 °C. The patient complained of general fatigue, which seemed to be unproportional to the physical stress caused by laparoscopic resection of tiny lesions. Fasting blood glucose level remained 80–90 mg/dl, which was slightly lower than that before the surgery. His serum sodium and potassium levels were within the normal ranges. On the 4th postoperative day, laboratory examination for irAE was performed, and prednisolone (100 mg/day) was empirically started for possible adrenal insufficiency. His body temperature and fasting blood glucose levels promptly returned to within a normal range, and general fatigue disappeared. Laboratory data on the 4th postoperative day revealed hypothyroidism (thyroid-stimulating hormone (TSH) 11.56 μIU/ml [reference value: 0.50–5.00 μIU/ml], Free-T3 0.94 pg/ml [reference value: 2.30–4.30 pg/ml], Free-T4 0.94 ng/dl [reference value: 0.90–1.70 ng/dl]) and hypopituitarism (adrenocorticotropic hormone (ACTH) < 1.5 pg/ml [reference value: 7.2–63.3 pg/ml]), Cortisol 2.75 μg/dl [reference value: 6.24–18.00 μg/dl]). Considering the transient hyperthyroidism during lenvatinib plus pembrolizumab administration (Fig. 3), we diagnosed hypothyroidism and hypopituitarism caused by lenvatinib plus pembrolizumab.

After the diagnosis of combined hypothyroidism and hypopituitarism, he received hydrocortisone (40 mg/day) first on the 9th postoperative day. Then, his fever, general fatigue, or any abnormality of fasting blood glucose level dissolved, and he was discharged on the 12th postoperative day. Following recovery of adrenal insufficiency, he received levothyroxine on the 14th postoperative day. He was alive without recurrence of gastric cancer and his laboratory data for thyroid and pituitary function remained stable while receiving hydrocortisone and levothyroxine for 2 years and 5 months.

## Discussion

In this case report, we present a patient who underwent laparoscopic hepatectomy after receiving lenvatinib plus pembrolizumab and developed hypothyroidism and hypopituitarism after surgery. Although many clinical trials of ICI have been conducted [5–7], few studies of irAEs after surgery have been reported. Given that endocrine irAEs, such as hyperthyroidism, hypothyroidism, and hypopituitarism were observed in several trials even after the discontinuation of ICI [8], it is important to consider the possible occurrence of irAEs when patients undergo surgery after receiving ICI. Although there have been only one case report in which inflammatory process was developed in the left parietal lobe in the brain as irAEs of nivolumab and ipilimumab after craniotomy for resection of metastatic tumor from renal cancer [9], to the best of our knowledge, the present case report is the first one to report the development of endocrine irAEs in the immediate postoperative period.

The incidence of endocrine irAEs ranges from 5 to 20% [10–12]. The incidence of thyroid dysfunction, such as hypothyroidism and hyperthyroidism, is reported to range from 6 to 20%. Typically, thyroiditis caused by ICI triggers transient hyperthyroidism, followed by transient or permanent hypothyroidism in a median of 6–12 weeks [13, 14]. In the present case, transient hyperthyroidism was self-limiting and TSH levels slightly increased just before surgery (5.93 µIU/ml), although Free-T3/T4 levels were within the normal range at that time (2.36 pg/ml and 0.91 ng/dl for Free-T3/T4, respectively). Although TSH was rising just before surgery, which might have been the early sign of irAE, we did not consider at that moment that it would provoke severe irAE immediately after surgery, because there had been no such reports. Now, our case indicates that even slightly elevated level of TSH needs attention before surgery. We consider that once the elevation of TSH is noticed, the initiation of thyroid hormone replacement therapy should be considered [15, 16]. Hypopituitarism, which leads to pituitary–adrenal insufficiency caused by ICIs, is also frequently observed. The frequency of hypopituitarism induced by anti-PD-1 antibody and ipilimumab was reported as < 1% and 10–17%, respectively [17]. Although the median time to hypopituitarism has been reported to range from 9 to 9.5 weeks [18, 19], Otsubo et al. reported that late-onset irAEs might occur several months after ICI therapy [20]. Current guidelines require the replacement of deficient hormones to manage hypopituitarism [21]. Although high incidences of hypothyroidism and hypopituitarism have been reported with the co-therapy of nivolumab and ipilimumab, it is rare that hypothyroidism and hypopituitarism occur at the same time by the treatment of single ICI [22]. In the present case, corticosteroid replacement was started before levothyroxine replacement was initiated. Without corticosteroid replacement, levothyroxine replacement may unmask or exacerbate adrenal insufficiency and trigger an adrenal crisis, because levothyroxine may increase the clearance of cortisol [23, 24]. In addition, we consider that surgical stress might exacerbate the development of hypopituitarism in this patient Indeed, Hahner et al. reported that hypopituitarism was mainly precipitated by stressful events, such as surgery [25].

It is well known that patients who respond to ICI tend to develop irAEs [26]. In the present case, the patient responded effectively to ICI therapy before surgery. In case the surgery is performed for patients with a good response to anti-cancer drugs, it is necessary to pay special attention to irAEs in patients with ICI in the postoperative period by checking general appearance and laboratory testing.

## Conclusions

We present a case of laparoscopic hepatectomy after receiving lenvatinib plus pembrolizumab and developed hypothyroidism and hypopituitarism after surgery. Regarding surgery after ICI therapy, it is important to recognize that irAEs might occur in the immediate postoperative period.

## Data Availability

All data supporting this study are included in this manuscript.

## Abbreviations

ICIs: Immune checkpoint inhibitors
irAEs: Immune-related adverse events
CTCAE: Common Terminology Criteria for Adverse Events
TSH: Thyroid-stimulating hormone
ACTH: Adrenocorticotropic hormone
